# Electrical gate control of spin current in van der Waals heterostructures at room temperature

**DOI:** 10.1038/ncomms16093

**Published:** 2017-07-05

**Authors:** André Dankert, Saroj P. Dash

**Affiliations:** 1 Department of Microtechnology and Nanoscience, Chalmers University of Technology, SE-41296 Göteborg, Sweden

## Abstract

Two-dimensional (2D) crystals offer a unique platform due to their remarkable and contrasting spintronic properties, such as weak spin–orbit coupling (SOC) in graphene and strong SOC in molybdenum disulfide (MoS_2_). Here we combine graphene and MoS_2_ in a van der Waals heterostructure (vdWh) to demonstrate the electric gate control of the spin current and spin lifetime at room temperature. By performing non-local spin valve and Hanle measurements, we unambiguously prove the gate tunability of the spin current and spin lifetime in graphene/MoS_2_ vdWhs at 300 K. This unprecedented control over the spin parameters by orders of magnitude stems from the gate tuning of the Schottky barrier at the MoS_2_/graphene interface and MoS_2_ channel conductivity leading to spin dephasing in high-SOC material. Our findings demonstrate an all-electrical spintronic device at room temperature with the creation, transport and control of the spin in 2D materials heterostructures, which can be key building blocks in future device architectures.

## Abstract (web summary)

Two-dimensional materials are unique to build heterostructures with contrasting spintronic properties. Here, Dankert and Dash utilize a van der Waals heterostructure with graphene and MoS_2_ to demonstrate an all-electrical device for creation, transport and control of the spin current up to room temperature.

Spintronics aims to exploit the spin degree of freedom in solid state devices for data storage and information processing technologies^1^,^2^. The fundamental spintronic device concepts, such as creation, manipulation and detection of spin current has been demonstrated in semiconductors^3^,^4^,^5^ and spin transistor structures^6^,^7^,^8^,^9^ using both electrical and optical methods. However, an unsolved challenge in the field is the realization of all-electrical methods to control the spin current and spin lifetime at ambient temperature^2^. For this purpose, two-dimensional (2D) crystals offer a unique platform for spintronics due to their remarkably broad range of spin-dependent properties^10^,^11^,^12^. Graphene has been demonstrated as an excellent material for spin transport and spin logic devices due to the low spin–orbit coupling (SOC) and high electron mobility^11^,^12^,^13^,^14^,^15^,^16^,^17^,^18^,^19^,^20^. However, this low SOC causes a big challenge for the electrical control of the spin polarization in graphene^11^,^12^. In contrast, transition metal di-chalcogenides (TMDCs) are semiconductors with a high SOC, several 10 meV in the conduction band and 100 meV in the valence band, which are orders of magnitude higher than in graphene^11^,^21^. Recently, such high-SOC TMDCs also showed fascinating spin dynamics and transport properties^22^,^23^,^24^,^25^.

To harvest these novel and contrasting spintronic properties of both 2D materials, hybrid devices, consisting of graphene and TMDC van der Waals heterostructures (vdWh)^26^,^27^, are proposed to be promising^28^. The proximity-induced strong SOC of graphene/TMDC vdWhs led to the observation of weak anti-localization^29^,^30^ and spin Hall effect^31^. In addition to the advantage of spin transport in graphene, SOC in such vdWhs can be utilized to electrically control the spin polarization. Recently, a spin field-effect switch has been demonstrated by using non-local (NL) spin-valve measurements in such a vdWh up to 200 K (ref. 32). For comprehensive understanding and advancement of 2D materials vdWh-based spintronic devices, demonstration of electrical gate-tunable spin current and spin lifetime by both spin-valve and Hanle spin precession measurements at ambient temperatures is a crucial requirement.

In this article, we demonstrate the electronic gate control of the spin current and spin lifetime at room temperature by using both spin-valve and Hanle measurements on graphene/MoS_2_ vdWh devices. Hanle spin precession measurements unambiguously prove the spin-polarized transport and demonstrate the electrical gate control of the spin lifetime and diffusion length in such 2D material heterostructures at room temperature. We further prove that the gate-voltage tuning of the Schottky barrier at the graphene–MoS_2_ interface and MoS_2_ channel conduction facilitate the spin in graphene to interact and dephase efficiently in high-SOC material MoS_2_. Extensive spintronic and electronic measurements in different devices at various gate voltages, injection bias currents and temperatures enable us to understand the gate control of spin relaxation mechanism in such vdWhs.

## Results

### Device and electrical gate control of spin current

**Figure 1:**
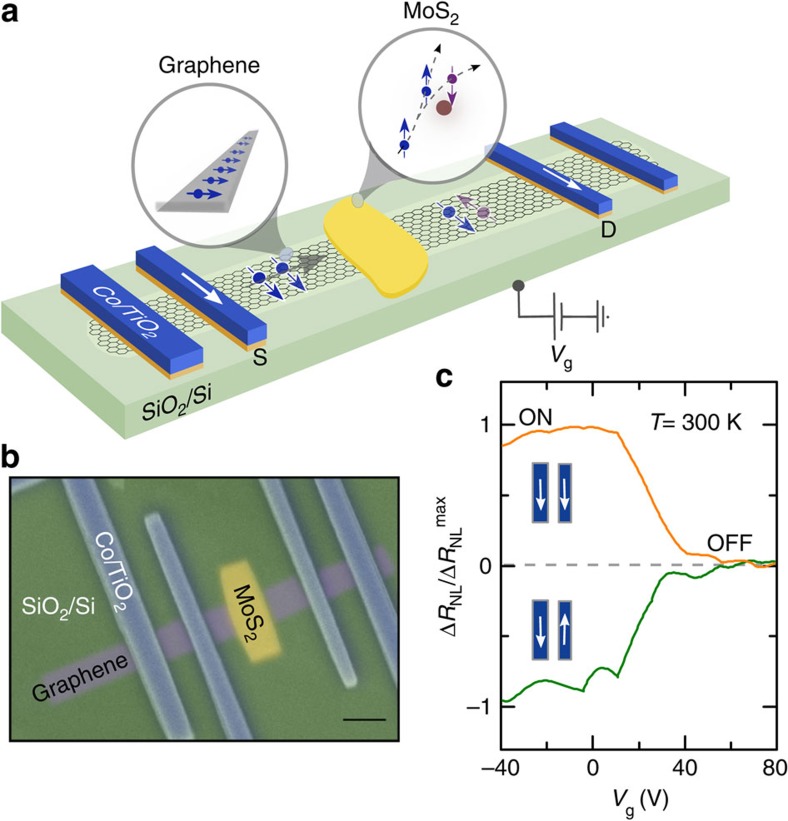
MoS_2_/graphene vdWh. (**a**) Schematics of graphene/MoS_2_ heterostructure channel with FM source (S) and drain (D) contacts. This structure allows spin injection into graphene from the source (S), diffusive spin transport in the graphene/MoS_2_ channel, spin manipulation by a gate voltage and detection of spin signal by the drain (D). (**b**) Coloured scanning electron microscope image of a fabricated device with a CVD graphene/MoS_2_ heterostructure channel and multiple FM tunnel contacts of TiO_2_(1 nm)/Co(80 nm) (scale bar, 1 μm). The devices are fabricated on Si/SiO_2_ substrate, which is used as a gate electrode for control of the spin polarization in the channel. (**c**) Gate dependence of the measured NL resistance 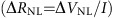 normalized to the maximum value, showing transistor-like ON/OFF spin signal modulation at room temperature, for parallel and antiparallel magnetization alignments of source and drain.

A schematic representation of a gate-controlled graphene/MoS_2_ vdWh spintronic device used for both spin-valve and Hanle measurements is shown in Fig. 1a. We fabricated the heterostructure devices with large-area chemical vapour deposited (CVD) graphene^13^ and TMDC molybdenum disulfide (MoS_2_) flakes^22^ (Fig. 1b). The spin current is generated and detected in NL measurement geometry by using ferromagnetic (FM) tunnel contacts deposited on graphene either in parallel or antiparallel magnetization configuration (see ‘Methods’ section)^13^. Our devices satisfy the four main requirements for a transistor-like spin signal modulation at room temperature:^33^ the electrical creation, diffusive transport, NL electrical detection and the crucial electrical control of a spin current by means of a gate voltage. Figure 1c shows the gate dependence of the measured spin signal modulation, demonstrating transistor-like ON/OFF states at room temperature. The measurements were performed by probing the NL voltage (Δ*V*_NL_) while sweeping the gate voltage (*V*_g_), for both parallel and antiparallel magnetization alignments of FM source and drain. To confirm such a gate-controlled spin device operations and to understand the spin relaxation mechanisms, we systematically investigated both the spin valve and Hanle spin precession measurements in graphene/MoS_2_ heterostructures.

### Electrical gate control of spin-valve signal

**Figure 2:**
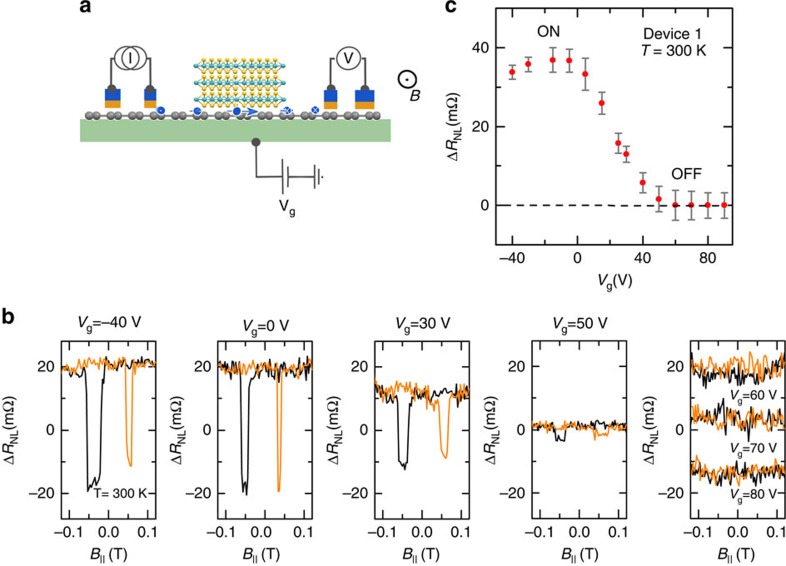
Gate-controlled spin valve signal at room temperature. (**a**) Schematic of the NL spin-valve measurement geometry, where the spin current injector circuit (I) and the voltage detector circuit (V) are placed in a NL geometry. The spin current diffusing in the heterostructure channel is detected as a voltage signal by the FM detector. The magnetization of the injector/detector FM contact and also the sign of spin accumulation are controlled by an in-plane magnetic field B_||_. (**b**) NL spin-valve magnetoresistance 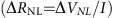 measurements at 300* *K by application of different gate voltages *V*_g_. Measurements are performed at a constant current source of *I=*30 μA. A NL linear background (few μV) due to stray charge current is subtracted from the signal. (**c**) Modulation of spin-valve signal magnitude Δ*R*_NL_ with gate voltage *V*_g_, showing ON/OFF states at 300* *K. The error bar is derived from the root mean square of the noise in the measured signal.

To investigate the spin transport behaviour in the graphene/MoS_2_ heterostructure channel, spin-valve measurements were performed in NL geometry at room temperature. Sweeping an in-plane magnetic field, the magnetization configuration of the injector and detector FM contacts (Fig. 2a) can be aligned parallel or antiparallel, resulting in a magnetoresistance Δ*R*_NL_. We observed a clear spin-valve signal (Fig. 2b) at room temperature, demonstrating the possibility of spin transport in the graphene/MoS_2_ heterostructures with a channel length of 2.6 μm and a width of 0.6 μm. The NL spin-valve signal 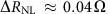 is smaller than usually measured in pristine CVD graphene channels of similar geometry^13^. This reduction could arise from SOC induced in the graphene channel in proximity with MoS_2_ (refs 28, 29, 30, 31).

Next, we measured the spin-valve signal at different gate voltages (*V*_g_) at room temperature (Fig. 2b,c). Below a threshold voltage *V*_g_<0 V, we observed spin-valve signals Δ*R*_NL_ with almost constant amplitude. For the gate-voltage range of 0<*V*_g_<50 V, a drastic reduction in Δ*R*_NL_ is observed, whereas for *V*_g_*>*50 V no spin signal could be detected. This behaviour reproduces the gate sweep NL resistance modulation behaviour we also measured in Fig. 1c. The strong modulation and vanishing spin current from ON to OFF state with application of gate voltage in our MoS_2_/graphene heterostructures indicates an electrical spin-control operation at room temperature. Spin-valve measurements with similar behaviour are recently reported at lower temperatures up to 200 K (ref. 32). The room temperature gate control in our spintronic devices can be attributed to better spin transport properties in graphene samples and less interfacial scattering. To further confirm the spin transport and to estimate the spin relaxation time, gate-dependent Hanle spin precession measurements in the graphene/MoS_2_ channel are necessary.

### Electrical gate control of spin lifetime

**Figure 3:**
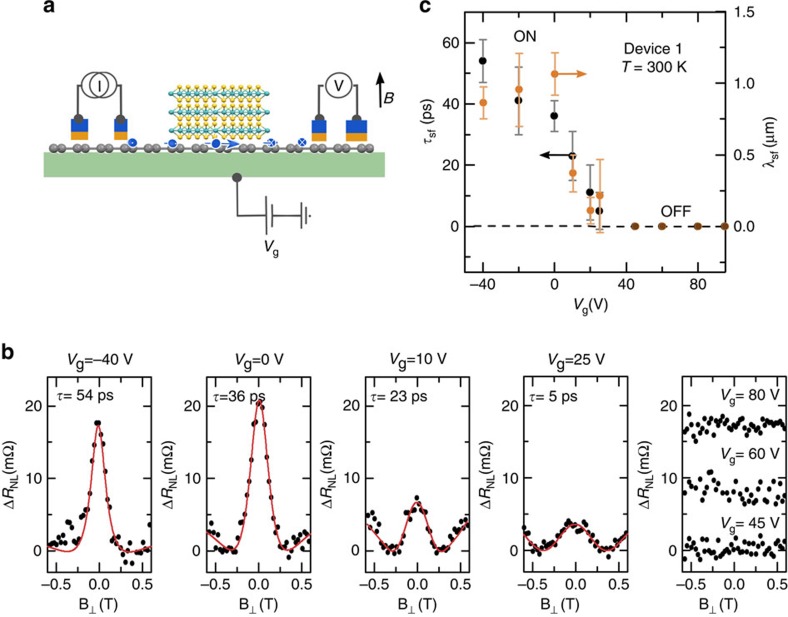
Gate-controlled Hanle spin precession at room temperature. (**a**) NL Hanle geometry, where source and drain magnetization are aligned parallel while sweeping a perpendicular magnetic field 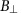. (**b**) NL Hanle spin signal 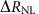 measured at 300* *K at different gate voltages. Measurements are performed at a constant current source of *I=*30 μA. The raw data points are fitted with equation (1) (red line) to extract spin lifetime *τ*_sf_ and diffusion length *λ*_sf._ (**c**) The gate voltage *V*_g_ dependence of spin lifetime *τ*_sf_ (black) and diffusion length *λ*_sf_ (orange) at 300* *K showing modulation of spin parameters from ON to OFF state. The error is derived from the error of the Hanle fit.

Consequently, we performed NL Hanle spin precession measurements in our graphene/MoS_2_ vdWh devices (Fig. 3a). Figure 3b shows the Hanle data measured for different gate voltages at room temperature. In this geometry, the modulation of the signal stems from the spin precession about a perpendicular magnetic field 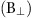 with the Larmor frequency 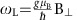 (Landé factor *g*=2)^34^,^35^. The variation of this NL resistance Δ*R*_NL_ due to precession and relaxation of the spins diffusing from the injector to the detector can be described by:


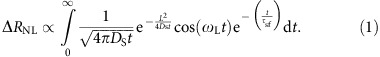


With the channel length L, we can extract the spin lifetime *τ*_sf_ and diffusion constant 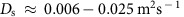 to calculate the spin diffusion length 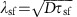 (Fig. 3c). Similar to the spin-valve measurements, we observed an almost constant Hanle signal for *V*_g_*<*0 V (Fig. 3b). In this range, we obtain a spin lifetime 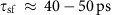 and diffusion length 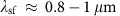 at room temperature. The reduced spin lifetime in our vdWhs, compared to pristine graphene devices (200–300 ps)^13^, indicates a proximity-induced SOC in graphene through MoS_2_. Such a shortened spin lifetime has also been expected using the spin–orbit relaxation time *τ*_so_ determined from spin Hall effect studies^31^, weak anti-localization measurements^29^,^30^ and theoretical predictions^28^,^32^. The 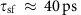 in our graphene in proximity with MoS_2_ corresponds to ∼1 meV of spin splitting at room temperature, which is gigantic compared to the few μeV in pristine graphene^28^. The induced SOC in graphene in proximity with MoS_2_ can be attributed to the hybridization of the carbon orbitals with the *d* orbitals of Mo (ref. 28). In the gate-voltage range of 0 V*<V*_g_*<*25 V, a strong tuning of the spin signal amplitude Δ*R*_NL_, spin lifetime *τ*_sf_ and consequently diffusion length *λ*_sf_ in the MoS_2_/graphene channel is observed. The *τ*_sf_ can be tuned down to ∼5 ps near *V*_g_*=*25 V. For *V*_g_*>*25 V, the Hanle signal could not be observed, validating a transition from ON to OFF state with gate voltage. This realization of a gate-controlled spin lifetime by an order of magnitude opens fascinating prospect for spin transport and spin relaxation in such graphene/TMDC hybrid systems.

The disappearance of the Hanle signal already at a gate voltage of about 30 V is due to the reduction of the amplitude by factor two compared to the spin-valve signal, which is consistent with the measurement principle (see Supplementary Fig. 1). Comparative measurements in a pristine graphene channel revealed only a weak modulation in Δ*R*_NL_ and *τ*_sf_ due to a change in carrier density around the Dirac point^13^. The observed strong modulation and vanishing spin signal in our vdWhs can originate from the gate-controlled interaction of spin-polarized electrons in graphene with high-SOC MoS_2_. This electrical control of spin signal is reproducibly observed over several devices fabricated on different chips and measured at various bias voltages, gate-voltage sweeps and temperatures (see Supplementary Figs 2–4).

## Discussion

To understand the gate-controlled spin modulation, we characterized the charge transport in both the MoS_2_ channel and the graphene/MoS_2_ vdWhs. The lateral MoS_2_ field-effect transistor shows a typical n-type transfer characteristic with an enhancement of current up to 10^6^ times with application of *V*_g_ at room temperature (see Supplementary Fig. 5). Such a gate-tunable MoS_2_ channel resistance in our MoS_2_/graphene vdWh would allow the tuning of a parallel spin transport channel next to graphene. If the spin current from graphene can enter MoS_2_ in the ON state, spins would lose their coherence at a much faster rate, due to the high SOC of MoS_2_.

**Figure 4:**
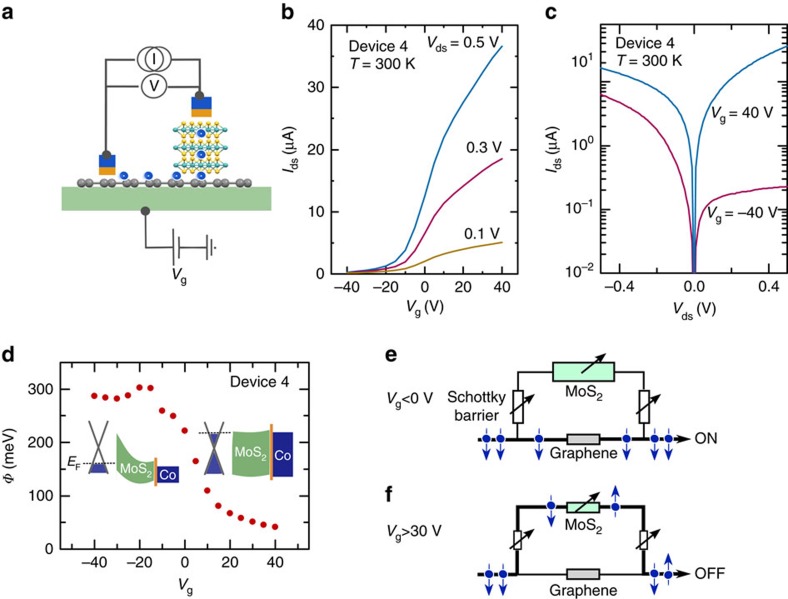
Tuning the Schottky barrier at the MoS_2_/graphene interface by gate voltage. (**a**) Schematics of the graphene/MoS_2_ vertical heterostructure. (**b**) Transfer characteristic (drain–source current *I*_ds_ versus gate voltage *V*_g_ for different drain–source voltages *V*_ds_) in a vertical device with MoS_2_ thickness of 35 nm. (**c**) Output characteristics (*I*_ds_ versus *V*_ds_) at 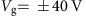. (**d**) Schottky barrier height *Ф* obtained for different *V*_g_. Inset: Band structures at the MoS_2_/graphene interface for *V*_g_<0 V and *V*_g_>30 V. (**e**) Representative circuit diagram of the graphene and MoS_2_ parallel transport channels connected by Schottky barrier resistors in the heterostructure. At gate voltages *V*_g_<0 V, the large Schottky barrier and high MoS_2_ resistance prevents spins from interacting with the high SOC MoS_2_ channel, resulting in a finite spin transport in graphene and corresponds to the spin-ON state. (**f**) At high gate voltages *V*_g_>30 V, the reduced Schottky barrier and MoS_2_ channel resistance allows spins to tunnel into MoS_2_ and hence dephasing in the high-SOC material, resulting in the spin-OFF state.

Next, we investigated the charge transport in vertical graphene/MoS_2_ devices, to understand the transport mechanisms at the interface (Fig. 4a). The transfer characteristic of such a device is typical for n-type field effect transistors with a current enhancement of more than 10^2^ times (ON/OFF) at room temperature (Fig. 4b). The corresponding output characteristic shows an asymmetric line shape in the OFF state, which becomes symmetric in the ON state (Fig. 4c). This transistor behaviour can be attributed to the presence of a gate-tunable Schottky barrier (Ф) at the MoS_2_/graphene interface^36^,^37^. We determined this barrier Ф from the temperature-dependent output characteristic using the thermionic emission model^22^,^36^ (see Supplementary Fig. 6). Figure 4d shows the gate dependence of Ф at the graphene/MoS_2_ interface, which changes from 300 to 50 meV when tuning the vertical transistor from OFF to ON state. This corresponds to a gate modulation of the graphene Fermi level with respect to the MoS_2_ conduction band as depicted in the insets of Fig. 4d. Consequentially, in the spin-ON state, a high Schottky barrier at the graphene/MoS_2_ interface and high-channel resistance of MoS_2_ prevents spins in the graphene to enter the MoS_2_ channel (Fig. 4e). In contrast, in the spin-OFF state, the Schottky barrier at the graphene/MoS_2_ interface is significantly reduced yielding an almost Ohmic contact, in addition to a drastic reduction in MoS_2_ channel resistance (Fig. 4f). Under these conditions, spins can easily enter MoS_2_, where they experience a much faster spin relaxation. This explains the reduction in spin lifetime in addition to the decrease in spin signal amplitude with gate voltage in our spin-modulation device. These findings demonstrate that the gate voltage can tune the band offset between materials having complementary spintronics properties, facilitating the spins in graphene to interact efficiently with MoS_2_.

In conclusion, the realization of the electrical control of spin current at room temperature using 2D material heterostructures is a significant step in the field of spintronics. Exploiting the complementary spintronic properties of graphene and MoS_2_ creates a unique platform, where the spin current can be tuned in a controlled manner by gate voltage. Combining the gate dependence studies of spin valve, Hanle spin precession and Schottky barrier control in graphene/MoS_2_ heterostructures, we experimentally demonstrate the remarkable control over the spin parameters. The all-electrical creation, transport and control of the spins in vdWhs also demonstrated an integration of novel gate-controlled spin device and non-volatile FM memory elements. The spin-ON state current can be increased by integrating FM source and drain contacts of Heusler alloys with high spin polarization^20^. The graphene/MoS_2_ spintronic devices can be further improved by introducing insulating hexagonal boron nitride (h-BN) as tunnel barrier for efficient spin injection^38^,^39^, for gate-controlled spin polarization via magnetic proximity effects^40^, h-BN as substrate for improved graphene properties^41^,^42^, and as ultra-thin gate dielectric for efficient gate control of the spin parameters in the channel. Our findings also open a new platform for the interplay of spin, charge and orbital degrees of freedom for testing a plethora of exotic physical phenomena. Consequently, novel electronic materials with tunable SOC in electron- and hole-doped regime^43^ and emerging materials with topological protection^44^ can be used in heterostructures with graphene to create novel spin phenomena and provide opportunities for new discoveries.

## Methods

### Device fabrication

The vdWhs were prepared using CVD graphene (Graphenea) on highly doped Si (with a thermally grown 285-nm-thick SiO_2_ layer) and cleaned by Ar/H_2_ annealing at 450 °C. The MoS_2_ flakes (single crystals from SPI supplies) were transferred on top of graphene, which was patterned into individual channels either before or after the MoS_2_ transfer. The graphene was patterned by photo- or electron beam lithography and oxygen plasma etching. Next, appropriate MoS_2_ flakes of ∼20–35 nm located on graphene were identified by an optical microscope for device fabrication. The contacts were patterned on graphene (and MoS_2_ flakes, in case of the vertical devices) by electron beam lithography. Finally, we used electron beam evaporation to deposit 8 Å Ti, followed by *in situ* oxidation in a pure oxygen atmosphere for 30 min, resulting in an about 1 nm thick TiO_2_ layer. Without interrupting the vacuum, we deposited 80 nm Co and finalized the devices by lift-off in warm acetone at 60 °C. In the final devices, the Co/TiO_2_ contacts on graphene act as source and drain for spin-polarized electrons, the MoS_2_/graphene heterostructure region used as the channel and the Si/SiO_2_ is used as a gate for manipulation of the spin polarization. A representative Dirac curve for the graphene vdWhs is shown in Supplementary Fig. 7.

### Measurements

The measurements were performed in a cryostat with variable temperature and magnetic field facility. The current is applied using a Keithley 6221 current source and the NL voltage is detected by a Keithley 2182A nanovoltmeter; the gate voltage was applied using a Keithley 2612 source metre.

### Data availability

The data that support the findings of this study are available from the corresponding authors on reasonable request.

## Additional information

**How to cite this article:** Dankert, A. & Dash, S. P. Electrical gate control of spin current in van der Waals heterostructures at room temperature. *Nat. Commun.* **8,** 16093 doi: 10.1038/ncomms16093 (2017).

**Publisher’s note**: Springer Nature remains neutral with regard to jurisdictional claims in published maps and institutional affiliations.

## Supplementary Material

Supplementary Information
