# Supplementary material for: Electrical gate control of spin current in van der Waals heterostructures at room temperature
Source: Nat Commun. 2017 Jul 5;8:16093. doi: 10.1038/ncomms16093 (PMC5504284; doi:10.1038/ncomms16093)
Supplement: Supplementary Information [file ncomms16093-s1.pdf]

File name: Supplementary Information
Description: Supplementary Figures and Supplementary References

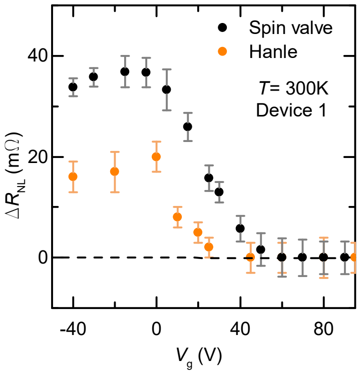

Supplementary Figure 1. **Electrical gate control of spin valve and Hanle signal at** 300 K. Comparison of spin signal amplitude of graphene/MoS_2 Device1 from non-local (NL) spin valve and Hanle measurements at room temperature, showing the electrical control of ΔR_{NL} with application of gate voltage (V_{g}). The spin valve signals measured by making parallel and anti-parallel alignment of ferromagnetic electrodes detect the full potential difference due to spin accumulation $\Delta\mu = \mu_{\uparrow} - \mu_{\downarrow}$, where $\mu_{\uparrow}$ and $\mu_{\downarrow}$ are electrochemical potentials for up- and down-spin, respectively. In comparison, the Hanle spin precession amplitude measured in the parallel alignment of ferromagnetic electrodes detects the half potential difference $\frac{1}{2}\Delta\mu = \mu_{\uparrow}$.[1,2] The error is derived by the root mean square of the noise of the signal.

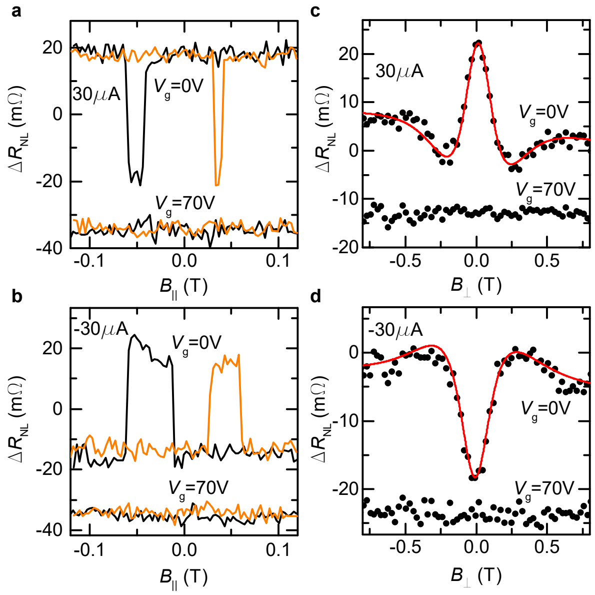

Supplementary Figure 2. **Spin signals at different bias currents and gate voltages in Device 1 at** 300 K. Non-local (NL) spin-valve magnetoresistance ΔR_{NL} measured at $V_{\mathrm{g}} = 0\,\mathrm{V}$ and $70\,\mathrm{V}$ at bias currents of **a.** $+30\,\mu\mathrm{A}$ and **b.** $-30\,\mu\mathrm{A}$. NL Hanle magnetoresistance ΔR_{NL} measured at $V_{\mathrm{g}} = 0\,\mathrm{V}$ and $70\,\mathrm{V}$ at bias currents of **c.** $+30\,\mu\mathrm{A}$ and **d.** $-30\,\mu\mathrm{A}$. The red line represents a NL Hanle fit according to Eq. 1 in the main paper. The data in the plots are shifted in Y-axis for clarity.

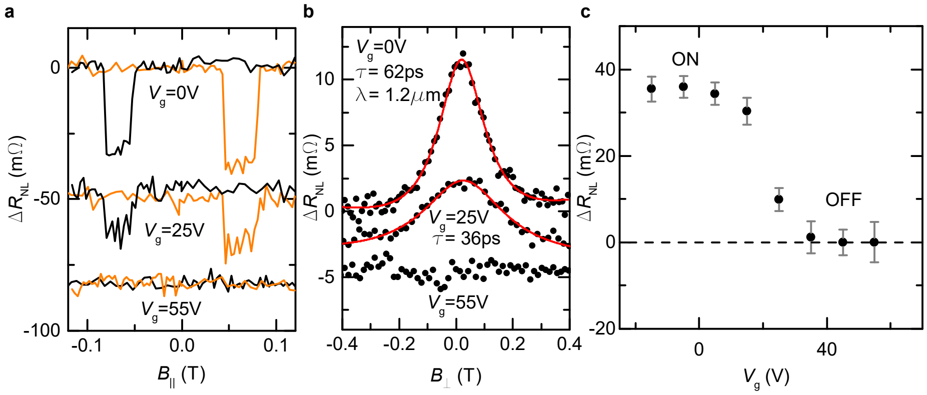

Supplementary Figure 3. **Electrical gate control of spin signal in Device 1 at** 75 K. **a.** Non-local (NL) spin-valve magnetoresistance ΔR_{NL} measured at 75 K at $V_{\mathrm{g}} = 0\,\mathrm{V}$ (ON-state), $V_{\mathrm{g}} = 25\,\mathrm{V}$ and $V_{\mathrm{g}} = 55\,\mathrm{V}$ (OFF-state). **b.** NL Hanle spin precession signal ΔR_{NL} measured at 75 K at $V_{\mathrm{g}} = 0\,\mathrm{V}$ (ON-state), $V_{\mathrm{g}} = 25\,\mathrm{V}$ and $V_{\mathrm{g}} = 55\,\mathrm{V}$ (OFF-state). The raw data points are fitted with the Hanle Eq.1 in the main paper, to extract spin lifetime τ_{sf}. The data in the plots are shifted in Y-axis for clarity. **c.** Spin signal modulation showing the control of the spin valve magnitude ΔR_{NL} with application of gate voltage (V_{g}) at 75 K. The error is derived by the root mean square of the noise of the signal. The spin signal amplitude and its gate dependent modulation at 75 K are found to be comparable with 300 K measurements. The Hanle spin precession measurements also resulted in similar spin lifetimes and diffusion lengths at 75 K. This indicates a weak temperature dependence of spin parameters in graphene/MoS_2 heterostructures.

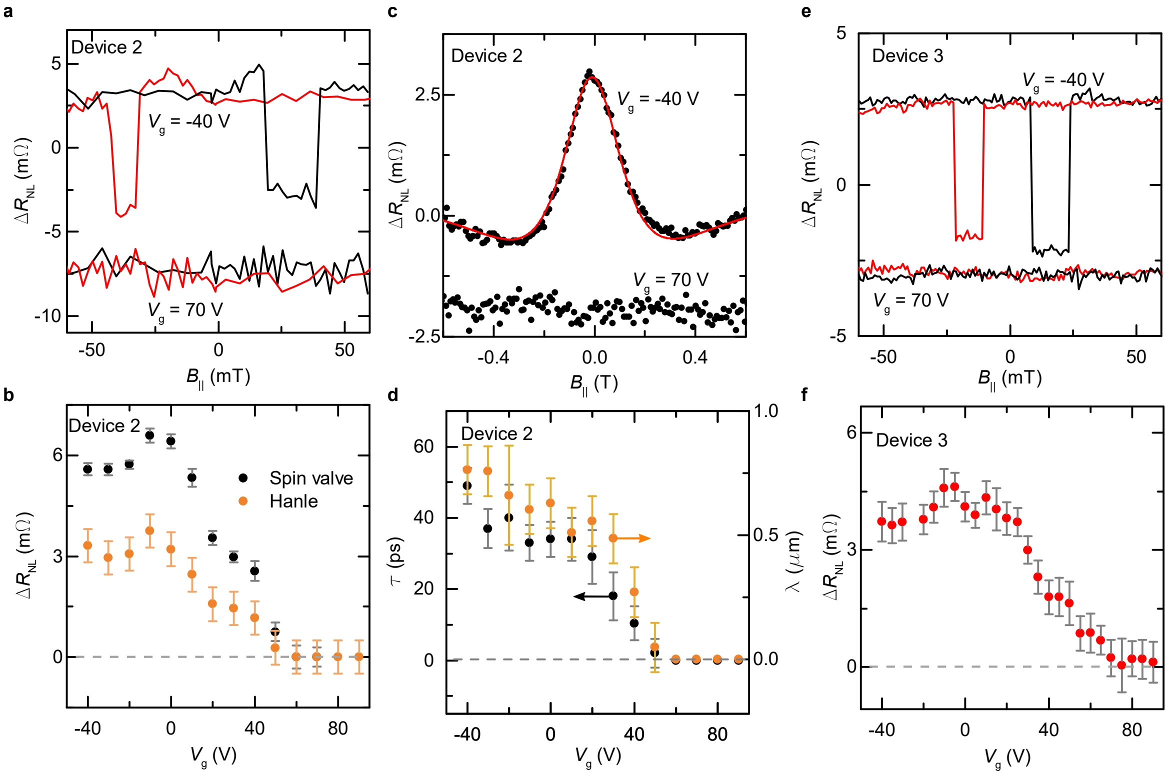

Supplementary Figure 4. **Reproducibility of electrical gate control of spin signal at room temperature.** Two more devices were fabricated and studied similar to the MoS_2/graphene heterostructure (Device 1) presented in the main text. **a.** Spin-valve signal measured in Device 2 at $V_g = -40\,V$ (ON-state) and $V_g = +70\,V$ (OFF-state). **b.** Gate control of spin signal amplitude from spin-valve and Hanle measurements in Device 2 at 300 K. The error is derived by the root mean square of the noise of the signal. **c.** Hanle spin precession signal measured in Device 2 at $V_g = -40\,V$ (ON-state) and $V_g = +70\,V$ (OFF-state). **d.** Gate control of spin lifetime and spin diffusion length extracted from the Hanle measurements in Device 2. The error is derived from the error of the Hanle fit. **e.** Spin-valve signal measured in Device 3 at $V_g = -40\,V$ (ON-state) and $V_g = +70\,V$ (OFF-state). **f.** Gate control of spin signal amplitude obtained from spin valve measurements in Device 3. The error is derived by the root mean square of the noise of the signal. Noticeably, the non-local spin resistance in Device 2 and 3 is significantly smaller compared to Device 1, however, the spin lifetime shows similar values. This is due to variations in tunnel spin polarization of ferromagnetic tunnel contacts prepared in different chips, which could be due to interfacial spin scattering.[3] These measurements confirm our observed behavior on Device 1 (presented in main manuscript) showing electrical gate control of spin parameters (magnitude of spin signal, spin lifetime and spin diffusion length) at room temperature and demonstrates a high reproducibility of our devices.

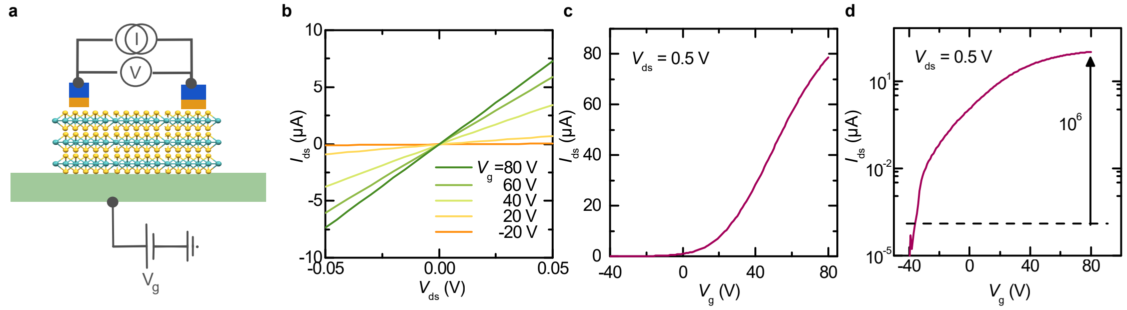

Supplementary Figure 5. **MoS_2 lateral device characterization at room temperature.** A lateral MoS_2 field effect transistor (FET) was prepared with Co/TiO_2 contacts (Device 5). **a.** Schematic of MoS_2 lateral device without a graphene bottom layer to study its intrinsic transistor performance in two-terminal measurement geometry. **b.** Output characteristics $I_{ds} - V_{ds}$ measured for different gate voltages (V_g) for MoS_2 channel with thickness of 30 nm. **c.** Transfer characteristic $I_{ds} - V_g$ with source-drain bias voltage $V_{ds} = 0.5\,V$ in a linear scaling. **d.** Transfer characteristic in logarithmical scaling typical for n-type MoS_2 FET devices with $I_{on}/I_{off} > 10^6$ and a field effect mobility of $\mu_f \approx 80\,cm^2\,(Vs)^{-1}$, which is similar to previous reports.[4,5] This shows that, in our graphene/MoS_2 heterostructures, MoS_2 provides a parallel conduction channel to graphene in the ON-state with application of gate voltage.

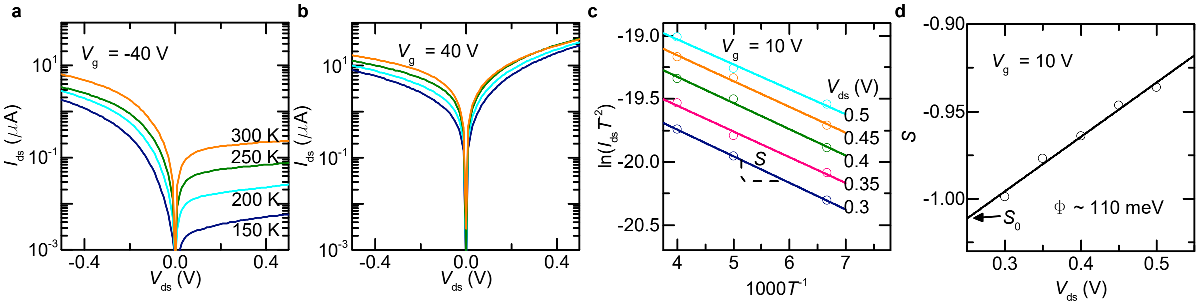

Supplementary Figure 6. **Schottky barrier at graphene/MoS_2 interface.** A vertical MoS_2/graphene heterostrucuture was prepared with Co/TiO_2 contacts (Device 4) to study the MoS_2/graphene interface properties (see also main text). We analyze the temperature dependence of the output characteristic $I_{\mathrm{ds}} - V_{ds}$ with different temperatures using the thermionic emission equation $I_{\mathrm{sat}} = AA^*T^2\exp\left(-\frac{e\Phi}{k_{\mathrm{T}}T}\right)$, where A^* is the Richardson constant, A is the contact area, e is the electron charge, k_{B} is the Boltzmann constant, and Φ is the Schottky barrier height. Output characteristics measured at different temperatures shown for **a.** $V_{\mathrm{g}} = -40\,\mathrm{V}$ and **b.** $V_{\mathrm{g}} = +40\,\mathrm{V}$. **c.** The Arrhenius plot $\ln\left(I_{\mathrm{ds}}T^{-2}\right)$ vs. T^{-1} for different bias voltages V_{ds} is shown exemplary for $V_{\mathrm{g}} = 10\,\mathrm{V}$ to extract the bias dependent slopes S. **d.** The extracted slopes S dependent linearly on V_{ds}: $S\left(V_{\mathrm{ds}}\right) = -\frac{e}{k_{\mathrm{B}}}\left(\Phi - V_{\mathrm{ds}}/n\right)$. The Schottky barrier Φ is evaluated from the extrapolated value of the slope at zero: $S_0 = S\left(0\right) = -\frac{e}{k_{\mathrm{B}}}\Phi$.

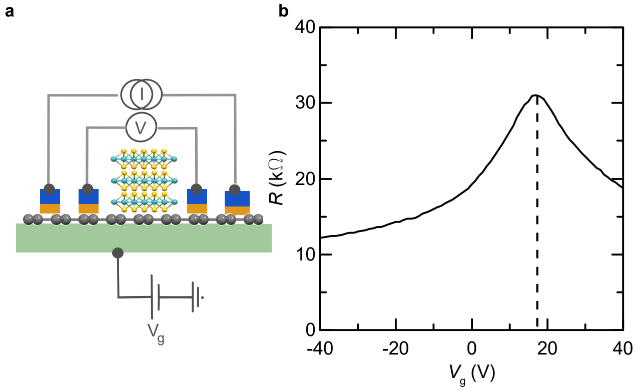

Supplementary Figure 7. **Channel resistance of graphene under MoS_2.** **a.** Schematic of the channel resistance measurement in graphene under a MoS_2 flake. **b.** Characteristic Dirac curve of graphene with a charge neutrality point around $V_{\mathrm{g}} = 16\,\mathrm{V}$ at $300\,\mathrm{K}$, indicating the good quality of the graphene is still preserved[1] after transfer of MoS_2. Such gate tuning of the graphene Fermi level helps in tuning the Schottky barrier at the graphene/MoS_2 interfaces. Consequently, this leads to the electrical control of spin polarization and spin lifetime in the MoS_2/graphene channel.

Supplementary References

[1]Kamalakar, M. V., Groenveld, C., Dankert, A. & Dash, S. P. Long distance spin communication in chemical vapour deposited graphene. *Nat. Commun.* **6**, 6766 (2015).

[2]Tombros, N., Jozsa, C., Popinciuc, M., Jonkman, H. T. & van Wees, B. J. Electronic spin transport and spin precession in single graphene layers at room temperature. *Nature* **448**, 571–574 (2007).

[3]Garzon, S., Zutić, I. & Webb, R. A. Temperature-dependent asymmetry of the nonlocal spin-injection resistance: evidence for spin nonconserving interface scattering. *Phys. Rev. Lett.* **94**, 176601 (2005).

[4]Dankert, A., Langouche, L., Kamalakar, M. V. & Dash, S. P. High-Performance Molybdenum Disulfide Field-Effect Transistors with Spin Tunnel Contacts. *ACS Nano* **8**, 476–482 (2014).

[5]Radisavljevic, B., Radenovic, A., Brivio, J., Giacometti, V. & Kis, A. Single-layer MoS2 transistors. *Nat. Nanotechnol.* **6**, 147–150 (2011).
